# Serum Metabolic Alterations upon Zika Infection

**DOI:** 10.3389/fmicb.2017.01954

**Published:** 2017-10-10

**Authors:** Carlos Fernando O. R. Melo, Jeany Delafiori, Diogo N. de Oliveira, Tatiane M. Guerreiro, Cibele Z. Esteves, Estela de O. Lima, Victoria Pando-Robles, Rodrigo R. Catharino, the Zika-Unicamp Network, Guilherme P. Milanez

**Affiliations:** Department of Genetic, Evolution and Bioagents, University of Campinas, Campinas, Brazil; Department of Genetic, Evolution and Bioagents, University of Campinas, Campinas, Brazil; Campinas Department of Public Health Surveillance, Campinas, Brazil; São Leopoldo Mandic Institute and Research Center, Campinas, Brazil; Clinical Pathology Department, Faculty of Medical Sciences, University of Campinas, Campinas, Brazil; Department of Genetic, Evolution and Bioagents, University of Campinas, Campinas, Brazil; Department of Genetic, Evolution and Bioagents, University of Campinas, Campinas, Brazil; Clinical Pathology Department, Faculty of Medical Sciences, University of Campinas, Campinas, Brazil; Obstetrics and Gynecology Department, School of Medical Sciences, University of Campinas, Campinas, Brazil; Obstetrics and Gynecology Department, School of Medical Sciences, University of Campinas, Campinas, Brazil; Obstetrics and Gynecology Department, School of Medical Sciences, University of Campinas, Campinas, Brazil; Obstetrics and Gynecology Department, School of Medical Sciences, University of Campinas, Campinas, Brazil; Clinical Pathology Department, Faculty of Medical Sciences, University of Campinas, Campinas, Brazil; Department of Genetic, Evolution and Bioagents, University of Campinas, Campinas, Brazil; Faculty of Food Engineering, University of Campinas, Campinas, Brazil; Clinical Pathology Department, Faculty of Medical Sciences, University of Campinas, Campinas, Brazil; Clinical Pathology Department, Faculty of Medical Sciences, University of Campinas, Campinas, Brazil; Faculty of Food Engineering, University of Campinas, Campinas, Brazil; Obstetrics and Gynecology Department, School of Medical Sciences, University of Campinas, Campinas, Brazil; Obstetrics and Gynecology Department, School of Medical Sciences, University of Campinas, Campinas, Brazil; Obstetrics and Gynecology Department, School of Medical Sciences, University of Campinas, Campinas, Brazil; Pediatric Immunology, Center for Investigation in Pediatrics, Faculty of Medical Sciences, University of Campinas, Campinas, Brazil; Pathology Department, Faculty of Medical Sciences, University of Campinas, Campinas, Brazil; Neurology Department, Faculty of Medical Sciences, University of Campinas, Campinas, Brazil; Department of Human Development and Rehabilitation, Faculty of Medical Sciences, University of Campinas, Campinas, Brazil; Campinas Department of Public Health Surveillance, University of Campinas, Campinas, Brazil; Department of Genetic, Evolution and Bioagents, University of Campinas, Campinas, Brazil; Department of Genetic, Evolution and Bioagents, University of Campinas, Campinas, Brazil; Department of Genetic, Evolution and Bioagents, University of Campinas, Campinas, Brazil; Department of Genetic, Evolution and Bioagents, University of Campinas, Campinas, Brazil; Department of Genetic, Evolution and Bioagents, University of Campinas, Campinas, Brazil; Department of Genetic, Evolution and Bioagents, University of Campinas, Campinas, Brazil; Department of Genetic, Evolution and Bioagents, University of Campinas, Campinas, Brazil; Department of Genetic, Evolution and Bioagents, University of Campinas, Campinas, Brazil; Department of Genetic, Evolution and Bioagents, University of Campinas, Campinas, Brazil; Department of Genetic, Evolution and Bioagents, University of Campinas, Campinas, Brazil; Department of Genetic, Evolution and Bioagents, University of Campinas, Campinas, Brazil; ^1^INNOVARE Biomarkers Laboratory, School of Pharmaceutical Sciences, University of Campinas, Campinas, Brazil; ^2^Unidad de Proteómica, Instituto Nacional de Salud Publica, Cuernavaca, Mexico

**Keywords:** Zika virus, mass spectrometry, metabolomics, viromics

## Abstract

Zika virus (ZIKV) infection has recently emerged as a major concern worldwide due to its strong association with nervous system malformation (microcephaly) of fetuses in pregnant women infected by the virus. Signs and symptoms of ZIKV infection are often mistaken with other common viral infections. Since transmission may occur through biological fluids exchange and coitus, in addition to mosquito bite, this condition is an important infectious disease. Thus, understanding the mechanism of viral infection has become an important research focus, as well as providing potential targets for assertive clinical diagnosis and quality screening for hemoderivatives. Within this context, the present work analyzed blood plasma from 79 subjects, divided as a control group and a ZIKV-infected group. Samples underwent direct-infusion mass spectrometry and statistical analysis, where eight markers related to the pathophysiological process of ZIKV infection were elected and characterized. Among these, Angiotensin (1-7) and Angiotensin I were upregulated under infection, showing an attempt to induce autophagy of the infected cells. However, this finding is concerning about hypertensive individuals under treatment with inhibitors of the Renin-Angiotensin System (RAS), which could reduce this response against the virus and exacerbate the symptoms of the infection. Moreover, one of the most abundant glycosphingolipids in the nervous tissue, Ganglioside GM2, was also elected in the present study as an infection biomarker. Considered an important pathogen receptor at membrane's outer layer, this finding represents the importance of gangliosides for ZIKV infection and its association with brain tropism. Furthermore, a series of phosphatidylinositols were also identified as biomarkers, implying a significant role of the PI3K-AKT-mTOR Pathway in this mechanism. Finally, these pathways may also be understood as potential targets to be considered in pharmacological intervention studies on ZIKV infection management.

## Introduction

Zika virus (ZIKV) was isolated for the first time in 1947 in the Zika forest, Uganda; a member of the Flaviviridae family, it is the etiologic agent of a disease with the same name, which is characterized as a self-limited infection where over 80% of the infected patients do not present any signs or symptoms (Duffy et al., 2009; Petersen et al., 2016). Individuals who present clinical manifestations of the disease usually develop unspecific symptoms such as fever, conjunctivitis, skin rashes, arthralgia, macular rash, myalgia, migraine, and retro-orbital pain, among other symptoms that may be clinically associated with the common influenza virus, as well as other arboviruses such as dengue (DENV), oropouche (OROV) or chikungunya (CHIKV) (Duffy et al., 2009; Daumas et al., 2013; Pabbaraju et al., 2016; Paniz-Mondolfi et al., 2016).

Because it was considered a relatively harmless infection up to 2014, ZIKV was not remarkably relevant in public health worldwide, remaining relatively unknown among people and even physicians. However, in view of the growing cases of microcephaly in newborns from ZIKV-infected mothers, a close relationship between the infection and problems during neural development has been established (Petersen et al., 2016). The result is a clinical condition characterized by abnormal brain development and decreased head diameter compared to individuals born from non-ZIKV-infected mothers. Additionally, patients suffering from this condition present impaired neurological functions, as well as delayed development of motor, speech, and cognitive functions (Woods et al., 2005). ZIKV has also been associated with an increased number of Guillain-Barre syndrome (GBS), an autoimmune disorder where the immune system attacks parts of the nervous system, resulting in acute (or subacute) flaccid paralysis due to nerve inflammation (Cao-Lormeau et al., 2016). Because of the severity of these events associated with ZIKV infection, the control of its main vector, mosquitoes from the *Aedes* genus (Petersen et al., 2016), has emerged as an important public health issue, given the difficulty in controlling its proliferation, especially in developing countries (Morrison et al., 2008; Bhatt et al., 2013; Boeuf et al., 2016). Moreover, the recent possibility of ZIKV transmission sexually and via hemoderivatives (Musso et al., 2015; Center for Biologics Evaluation and Research, 2016; Fréour et al., 2016; Katz and Rossmann, 2016; Russell et al., 2016) has created a context in which understanding the pathophysiological mechanism of infection became vitally relevant to pave the way toward the development of effective therapies, and to prevent associated aggravations.

For all these reasons, better understanding the pathophysiological mechanism of diseases is critical for delivering improved patient care. Recent advances in analytical approaches and metabolomics studies have been growing in the last few years and expanded the knowledge physiological and pathological alterations in living organisms (Dunn et al., 2013; Junot et al., 2014; Melo et al., 2016b). In line with this trend, this contribution focuses in understanding metabolomic alterations caused by ZIKV infection in serum samples from patients infected with ZIKV.

Recent literature states that there are important alterations in human cell metabolome (lipidome) caused by flaviviruses (Martín-Acebes et al., 2016). Such as alterations in the biosynthesis of steroid hormones and fatty acids, catabolism of phospholipids, and β-oxidation (Cui et al., 2013). In DENV-infected mosquitoes, for example, alterations of circa 15% on cell lipidome are observed when compared to uninfected cells. These alterations happen mostly on cell membranes, and correspond to up to 85% of the existing lipid species (Perera et al., 2012), and the nature of these alterations was corroborated by a previous contribution from our group in ZIKV-infected mosquito cells (Melo et al., 2016a). Thus, lipid metabolites have become a promising molecular class, still little explored in the pathophysiological mechanisms of disease and infection, where they have shown capabilities of associating prognostic and diagnostic of infections (van Gorp et al., 2002; Durán et al., 2015; Lima et al., 2015). This report ultimately aims at verifying serum lipid metabolites alterations in ZIKV-infected patients using direct infusion high-resolution mass spectrometry.

## Materials and methods

### Ethics statement

This study was conducted according to the principles expressed in the Declaration of Helsinki and was approved by the Ethics Committee of Unicamp (CEP-Unicamp: Comitê de Ética em Pesquisa da Unicamp—Campus Campinas), number 053407/2016. A written informed consent was obtained from all patients prior to enrollment. All samples were obtained from the Clinical Hospital of the University of Campinas.

### Research participants

#### Study design and rationale

This study included 79 subjects, regardless of age and gender, divided into a control group and a ZIKV group. The ZIKV group was composed of individuals that were positive after testing with the gold standard methodology for detecting ZIKV infection: real-time reverse transcription polymerase chain reaction (RT-PCR) (Lanciotti et al., 2008). According to the results obtained from RT-PCR, samples were treated as either RT-PCR(+) or RT-PCR(–) for ZIKV; all positive samples for ZIKV were also screened for other arboviruses to ensure the absence of cross-infections. On the other hand, for the control group to be considered heterogeneous and faithful to a “real-world” condition, in addition to including healthy individuals and symptomatic patients that were negative for ZIKV according to RT-PCR, we also did not perform testing for any other pathogens. This was to ensure that any biomarkers elected further in the study would pertain to ZIKV infection only, thus providing an unbiased metabolomic result. A summary of subject selection with the three subgroups rendered can be found below.

#### ZIKV-infected patients—RT-PCR(+), ZIKV group

The group of symptomatic patients, whose RT-PCR test was positive for ZIKV infection; it corresponded to 35 adult patients, which also presented clinical features compatible with ZIKV infection (i.e., fever, joint pain, conjunctivitis, and rash).

#### Symptomatic patients—RT-PCR(–), control group

A group of symptomatic patients, whose RT-PCR test for ZIKV was negative; it corresponded to 34 patients, which presented the same clinical features described above for ZIKV the group.

#### Healthy individuals—RT-PCR(–), control group

The control group was composed by 10 healthy adults, i.e., asymptomatic individuals who did not present any signs of infection within 30 days prior to sample collection and, therefore, presented a negative result in RT-PCR for ZIKV.

The collected specimens from all participants of the present study consisted of blood (serum) samples. Table 1 organizes the structure of sample collection and provides a view of the total number of analyzed specimens, according to type and group. All RT-PCR were performed using RNA extracted from the serum of the analyzed subjects.

**Table 1:** Demographics and clinical conditions of all recruited and included individuals in the study.

**Parameters**	**Groups**		
	**Control**		**ZIKV**
RT-PCR exam	Negative	Negative	Positive
Symptomatic?	No	Yes	Yes
**DEMOGRAPHICS**			
Male	6	25	27
Female	4	9	8
Mean age (median)	32.76 (30)	31.67 (30)	35.45 (35)
**SYMPTOMS**			
Fever (%)	NA^a^	17.14	29.40
Rash (%)	NA^a^	20.00	41.18
Joint pain (%)	NA^a^	2.86	11.76
Retro-orbital pain (%)	NA^a^	5.71	5.88
Migraine (%)	NA^a^	8.57	17.60
Conjunctivitis (%)	NA^a^	14.29	17.60
Neurological syndrome (%)	NA^a^	17.14	8.80

a *NA, Not Applicable*.

### PCR diagnosis

In order to confirm ZIKV infection, the viral stock and sample suspects of ZIKV-infected were assayed by real time RT-qPCR (Lanciotti et al., 2008). Briefly, the viral RNA was isolated by a commercial kit following the manufacturer's instruction (RNeasy Mini Kit, Qiagen, Hilden, Germany). One-step RT-PCR amplification of viral RNA (Taqman RNA to-CT, Applied Biosystems) was performed with following primers and probes: ZIKV-F: 5′- CCGCTGCCCAACACAAG-3′; ZIKV-R: 5′- CCACTAACGTTCTTTTGCAGACAT−3′; ZIKV-P: 5′-/FAM/AGCCTACCTTGACAAGCAGTCAGACACTCAA/-3′. All reactions were assembled in a final volume of 12.5 μL with 300 ng of RNA, 1× PrimeTime mix (Integrated DNA Technologies) containing both primers and probe, and 6.25 μL of TaqMan master mix (Applied Biosystems) by using the following cycling algorithm: 48°C for 30 min, 95°C for 10 min, followed by 45 cycles of 95°C for 15 s, and 60°C for 1 min.

### Sample preparation for HRMS

For sample preparation, 20 microliters of each biological sample (blood serum) were diluted in 200 μL of tetrahydrofuran and homogenized under vortex for 30 s; the volume was then completed to 1 mL with methanol, with further homogenization. The obtained solution was centrifuged for 5 min under 3,200 rpm. Twenty microliters of the supernatant was then collected and diluted in 980 μL of methanol, resulting in the final solution, which was divided in two 500-μL portions for analysis in positive and negative ion modes after the addition of 0.1% of formic acid and ammonium hydroxide, respectively.

### HRMS analyses

All samples were directly infused into an ESI-LTQ-XL Orbitrap Discovery (Thermo Scientific, Bremen, Germany) with a nominal resolution of 30,000 (FWHM). Data were acquired in the survey scan mode, according to the following parameters: flow rate at 10 μL.min^−1^, capillary temperature at 280°C, spray current at 5 kV, and sheath gas at 5 arbitrary units. Each sample was analyzed in quintuplicate. The utilized mass range for analysis was 700–1,800 m/z.

### Statistical analyses and structural proposals

Biomarkers choice was guided by using the orthogonal partial least squares discriminant analysis (OPLS-DA). Being a variation of the partial least squares discriminant analysis (PLS-DA), OPLS-DA is a supervised multivariate regression method that performs the linear combination of the original variables, thereby extracting, from raw mass spectrometry data, features that are responsible for sample grouping. The main difference of OPLS-DA from PLS-DA is that it uses orthogonal signal correction in order to maximize the explained covariance among the components of the model. For this analysis, interquartile range was used as data filtering method, with quantile normalization and range scaling. All analyses were performed using the online platform MetaboAnalyst 3.0 (Xia and Wishart, 2011, 2016).

After careful selection of the candidate markers using the statistical model, the significance of each ion was assessed by comparing signal intensities in the raw data matrix to ensure that all elected candidates (i) were above the signal-to-noise ratio and (ii) were not significantly present in the control group (intensities below the signal-to-noise ratio). For characterization, HMDB version 3.6 (Human Metabolome database—www.hmdb.ca), METLIN (Scripps Center for Metabolomics, La Jolla, CA), as well as Lipid MAPS online database (University of California, San Diego, CA—www.lipidmaps.org) were consulted to elect the most suitable marker. Mass accuracy was the method of choice for database research, with a maximum adopted mass error of 2 ppm.

## Results

**Figure 1:**
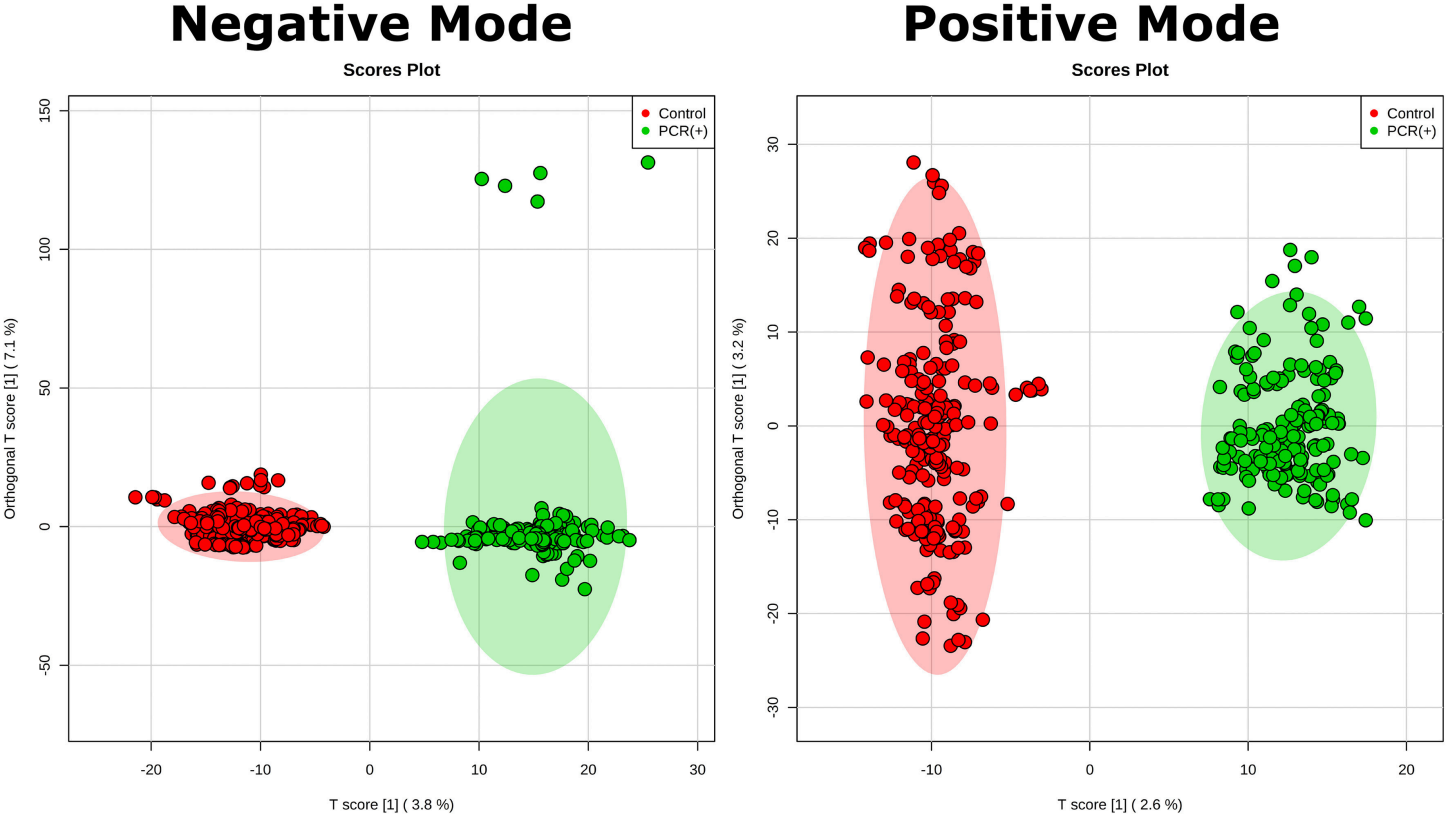
Establishment of the OPLS-DA model. The figure illustrates the score plot of OPLS-DA modeling for serum metabolomics data on positive and negative mode. The non-infected serum group clustered to the left region and the infected serum group clustered to the right area in the both positive and negative modes. The shaded area shows represents the confidence interval of 95% from OPLS-DA models; the T score [1] shows the relevance of the predictive component [1] in explaining the clustering model.

Statistical analysis was performed by using OPLS-DA, based on mass spectral data obtained by the direct infusion of serum, using the results from RT-PCR (absence or confirmation of ZIKV) to provide guidance and support in the establishment of the two studied groups. A detailed description with individuals' demographics and clinical conditions from each group is provided in Table 1. The rationale of mixing symptomatic patients and healthy individuals in the control group was a key feature of this study, as it is an ideal representation of the heterogeneity found in any given population in terms of clinical status. The absence of ZIKV infection in the symptomatic individuals of the control group, as determined by RT-PCR, increases the level of confidence in the biomarkers that were elected by the statistical modeling, thereby providing another level of assurance that such molecules are indeed related to ZIKV and no other related viral infection. This was ultimately corroborated when the OPLS-DA graph was plotted and the two groups remained isolated. In this sense, RT-PCR results were validated by OPLS-DA and vice-versa, as presented in Figure 1. The two-dimensional plot evidenced two very clear clusters, regardless of the ion mode analyzed in HRMS, grouping samples with similar ion content; the green cluster represents patients with RT-PCR-positive ZIKV infection, while the red cluster represents all other individuals that were negative for ZIKV after RT-PCR assessment.

**Figure 2:**
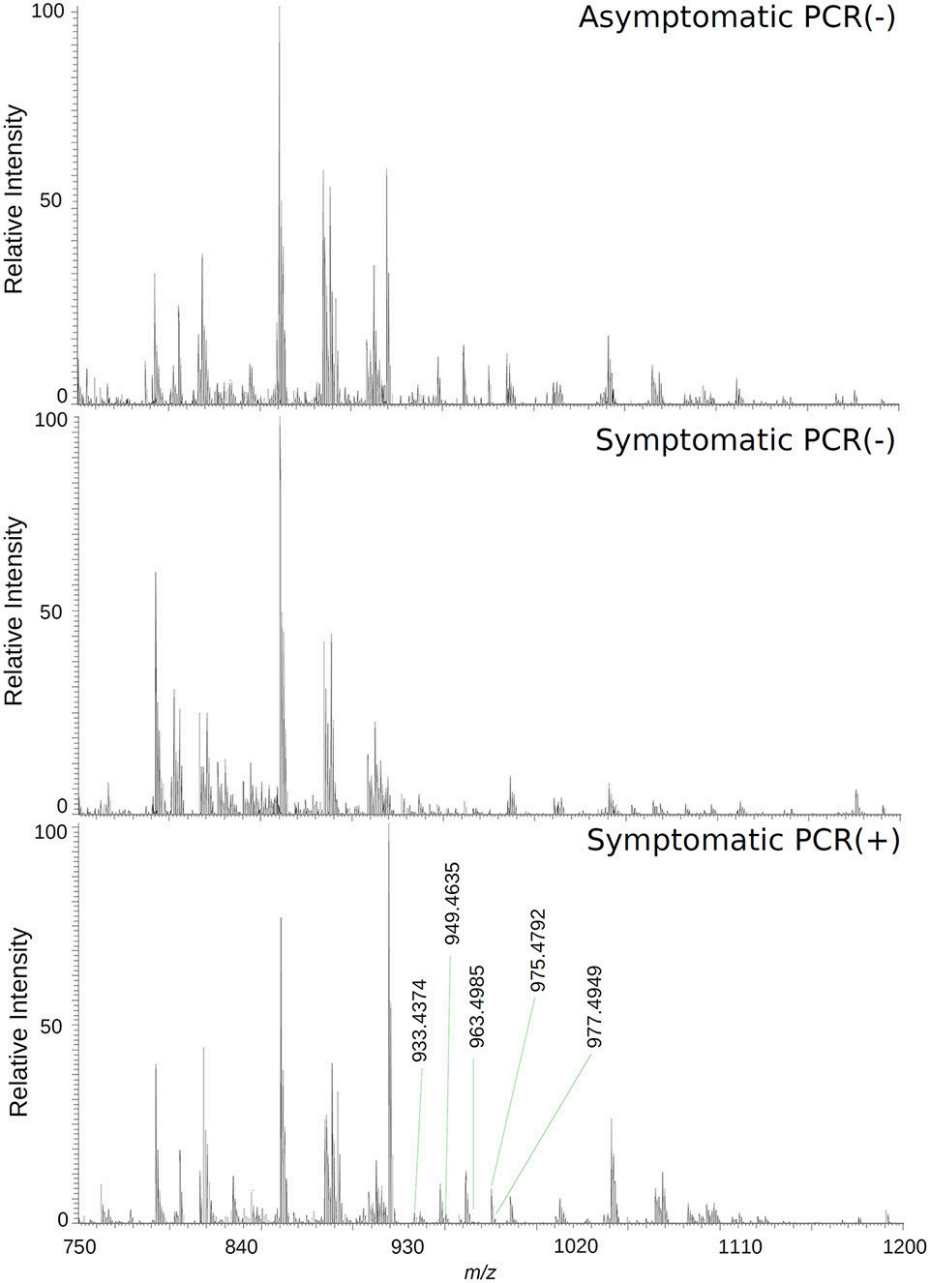
High-resolution mass spectrum of patients' serums on the negative ion mode: asymptomatic individuals with negative PCR results, patients with clinical manifestations of Zika virus infection and negative diagnosis by PCR, and patients with clinical manifestations of Zika virus infection and diagnosis Positive by PCR.

**Figure 3:**
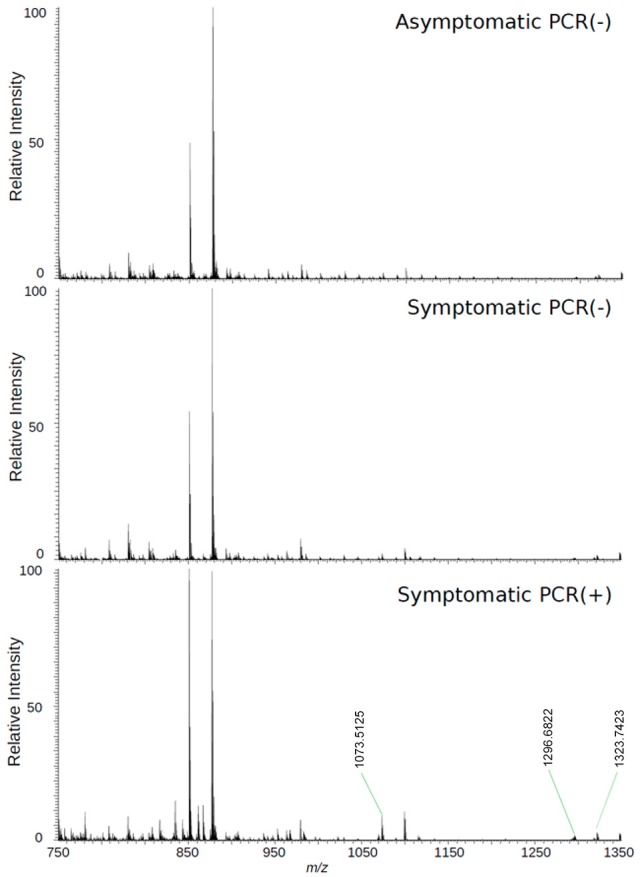
High-resolution mass spectrum of patients' serum on the positive mode: asymptomatic individuals with negative PCR results, patients with clinical manifestations of Zika virus infection and negative diagnosis by PCR, and patients with clinical manifestations of Zika virus infection and diagnosis Positive by PCR.

The loadings plot from the statistical model formed by features selected by OPLS-DA assisted in obtaining a list of ions (i.e., the candidate biomarkers) that were specific for the ZIKV group, according to the presented clustering model. Fifty major features were pointed out as the markers that described the ZIKV group in each ion mode on mass spectrometry (positive and negative), rendering one-hundred features in total. After crosschecking data from literature and metabolomics databases, three biomarkers were characterized for the positive mode: a phosphatidylinositol bisphosphate (PIP2), Angiotensin I, and Ganglioside GM2; for the negative mode, five biomarkers were identified and characterized: Angiotensin (1-7), and four phosphatidylinositol phosphates (PIP). A thorough description of all identified biomarkers is given in Table 2. It is important to remark that not only are the main selected ions supported by the statistical model, but also they are in line with the spectral data in each ion mode (Figures 2, 3).

**Table 2:** Lipid markers elected by OPLS-DA from serum analysis of patients infected with Zika Virus (ZIKV group).

**Exact mass**	**Theoretical mass**	**Error (ppm)**	**Adduct**	**ID^a^**	**Molecule**
**NEGATIVE MODE**					
977.4949	977.4929	2.04	[M+Cl]^−^	61356	PIP(18:1/18:1) and/or
				61365	PIP(18:2/18:0) and/or
				61384	PIP(20:2/16:0)
933.4374	933.4355	2.03	[M+Cl]^−^	71112	Angiotensin (1-7)
963.4985	963.5005	−2.07	[M-H]^−^	61399	PIP(20:4/18:1) and/or
				61395	PIP(20:3/18:2) and/or
				61403	PIP(20:4/18:1) and/or
				61319	PIP(16:0/22:5) and/or
				61405	PIP(22:3/16:2)
949.4635	949.4616	2.00	[M+Cl]^−^	61326	PIP(16:2/18:0) and/or
				61323	PIP(16:1/18:1) and/or
				61364	PIP(18:2/16:0)
975.4792	975.4772	2.05	[M+Cl]^−^	61366	PIP(18:2/18:1) and/or
				61374	PIP(18:3/18:0) and/or
				61386	PIP(20:3/16:0)
**POSITIVE MODE**					
1073.5125	1073.5103	2.04	[M+Na]^+^	61492	PIP2(20:0/18:2) and/or
				61495	PIP2(20:1/18:1) and/or
				61498	PIP2(20:2/18:0) and/or
				61423	PIP2(16:0/22:2)
1296.6822	1296.6848	−2.01	[M+H]^+^	65540	Angiotensin I
1323.7423	1323.7395	2.11	[M+Na]^+^	62596	Ganglioside GM2 (d18:0/12:0)

a *METLIN ID*.

**Figure 4:**
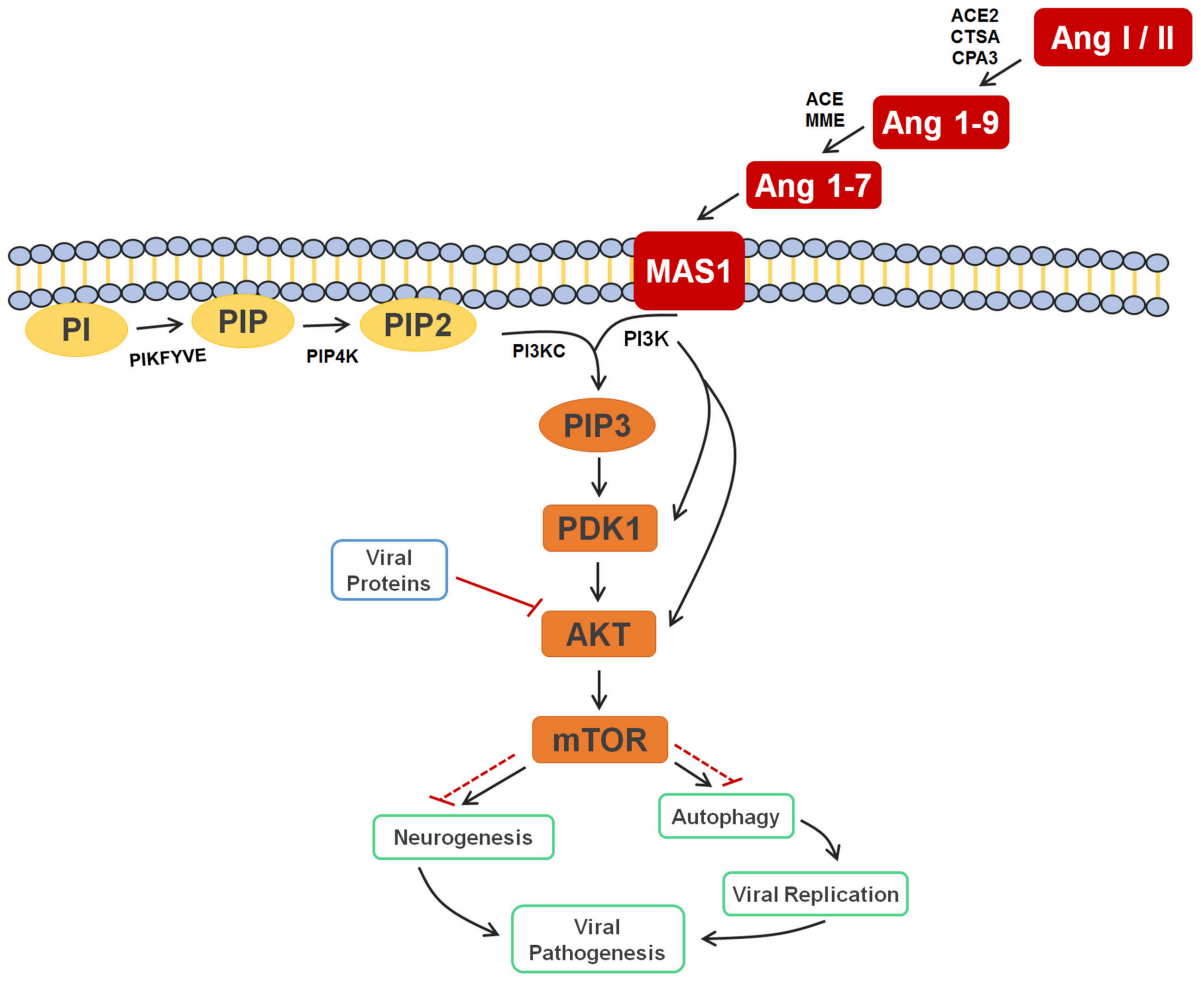
Cell signaling pathway scheme of metabolic alterations due to Zika virus infection. The scheme shows the cell response, attempting to control the viral infection, with Ang I or II, Ang 1-9, and Ang 1-7 signaling to activate the autophagy process, which would lead to cell death and, consequently, decreased viral replication. It is also possible to see the close participation of lipids PI, PIP2, and PIP3 as key players in this process, all of which were elected as biomarkers. The scheme also shows the inhibition of AKT by the viral proteins of ZIKV (solid and dashed red lines), which culminates in the inhibition of autophagy, so that replication can occur. In a parallel mechanism, it is possible to see that the same pathway is responsible for the inhibition of neurogenesis. Ang I/II, Angiotensin I/II; Ang 1-9, Angiotensin 1-9; Ang 1-7, Angiotensin 1-7; MAS1, MAS receptor; PI, 1-Phosphatidyl-D-myo-inositol; PIP, Phosphatidylinositol 5-phosphate; PIP2, Phosphatidylinositol-4,5-bisphosphate; PIP3, Phosphatidylinositol-3,4,5-trisphosphate; PDK1, 3-phosphoinositide-dependent protein kinase 1; AKT, AKT serine/threonine kinase 3; mTOR, mechanistic target of rapamycin (atypical serine/threonine kinase); PIKFYVE, 1-phosphatidylinositol-3-phosphate 5-kinase; PIP4K, phosphatidylinositol-5-phosphate 4-kinase type 2 alpha; PIK3C, phosphoinositide-3-kinase regulatory subunit 5; PIK3, phosphoinositide-3-kinase regulatory; ACE2, angiotensin-converting enzyme 2; CTSA, carboxypeptidase C; CPA3, carboxypeptidase A3; ACE, angiotensin-converting enzyme; MME, Neprilysin.

Finally, supported by literature information, we were able to provide the significance and the roles that all selected biomarkers play in a very particular metabolic pathway, the PI3K-AKT-mTOR. As displayed in Figure 4, these molecules were selected probably due to their accumulation after cell response to the blockade of AKT by viral proteins, thereby inhibiting relevant mTOR-related mechanisms such as autophagy and neurogenesis, and providing evidence that ZIKV infection has a very close relationship with the renin-angiotensin system (RAS).

## Discussion

Biomarker elucidation has revealed the presence of Angiotensin (1-7) (Ang (1-7) [m/z 933.4355] in the negative ion mode (Figure 2), and Angiotensin I (Ang I) [m/z 1296.6848] in the positive ion mode (Figure 3). These two metabolites are part of the RAS, which is directly involved in the uptake and excretion of sodium and potassium; these two ions promote vasoconstriction and blood pressure regulation, respectively (Tikellis et al., 2011; Passos-Silva et al., 2013). RAS has always been studied with focus on its role in metabolic syndromes such as obesity and hypertension (Van Vark et al., 2012; Santos et al., 2013; Cabandugama et al., 2017); however, its importance in the viral infection process has only recently been observed, as demonstrated by a study in which DENV-infected rats were treated with either losartan or enalapril. In this case, the treatment decreased DENV absorption by macrophages, showing that the RAS may be associated with infection severity (Hernández-Fonseca et al., 2015). In an experiment carried out with knockout rats for the angiotensin II converting enzyme (ACE2) and wild-type rats, both infected by the respiratory syncytial virus (RSV), knockout rats presented a five-fold higher viral titer than wild-type rats, in addition to increased pulmonary injury, mortality and angiotensin II (Ang II) plasma concentrations (Gu et al., 2016). In another study, also carried out with knockout rats for ACE2, this time assessing the infection by H5N1 virus (avian flu), has also demonstrated that knockouts presented a more severe infection, as well as higher mortality compared to wild-type animals. This result was, therefore, associated with increased plasma levels of Ang II, which presented close relationship with the severity and lethality of the avian flu (Zou et al., 2014). This was also observed for the H7N9 virus infection, where knockout rats for ACE2 presented increased plasma levels of angiotensin II, also with increased infection severity. The lack of ACE2 results in deficiency of Ang (1-7), a cleavage product of either angiotensin I or angiotensin II that is highly dependent on ACE2 activity to be formed (Lumbers and Pringle, 2014). Since Ang (1-7) has been associated with infection mitigation, its absence in knockout animals for ACE2 may be directly related to the severity of viral infection (Ferrario and Iyer, 1998).

Given that Ang (1-7) diminishes the severity of pathogen infections due to alterations in the cell machinery, thereby breaking its life cycle (Saraiva et al., 2011; Fedson, 2016), the biomarkers elucidated in our study reveal that these species may also be linked to the control of the immune response to ZIKV infection. One of the most primordial forms of innate immune defense may be autophagy, which has been described as a mechanism involved with antigen presentation, microbe elimination, and secretion of immune mediators (Tallóczy et al., 2006; Deretic et al., 2013). The Ang (1-7) signaling pathway, for instance, is also related to the process of autophagy, a process that causes infected cells to die, thus decreasing viral replication rate in the organism (Saraiva et al., 2011; Petersen et al., 2016). Electing both Ang I and Ang (1-7) as ZIKV group biomarkers, therefore, allow us to infer that the RAS is part of the immune response process against ZIKV in humans.

The other four markers that were found for ZIKV-infected patients are phosphatidylinositol phosphates (Table 2); these help corroborate the role of Ang (1-7) in the immune response upon infection, since this peptide is responsible for activating the PI3K-AKT-mTOR pathway (Giani et al., 2007; Sampaio et al., 2007). This process initiates a series of lipid phosphorylations upon binding to the MAS receptor, which modulates the activation of PI3K and leads to the activation of the phosphatidylinositol signaling system (PSS). The activation of PSS is also part of the cell signaling system for autophagy, hence the importance of PIPs on immune response during the infection. Although both the RAS activation and autophagy process contribute with higher biomarkers concentrations, an additional factor seem to cause the increase of the elected markers in the viral infection process of ZIKV. As demonstrated in the metabolic scheme presented in Figure 4, two non-structural ZIKV proteins, NS4A and NS4B, inhibit the AKT-mTOR signaling pathway (Liang et al., 2016). Such inhibition leads to the accumulation of intermediate metabolites and precursors involved in the PI3K-AKT-mTOR pathway signaling. As the virus inhibits AKT and the signaling through the RAS (Ang (1-7)) persists due to viral infection, the positive modulation over PI3K is maintained. Therefore, ZIKV infection has induced alterations in different signaling pathways, which have culminated with the overexpression of some metabolites, amongst them the above-reported lipids.

This is the first time that lipids for ZIKV infection are described, whereas the great majority of previous contributions deal with general immune response species such as proteins, as well as with molecules potentially linked with microcephaly (Petersen et al., 2016). Taking into account that neurologic malformations such as microcephaly are associated with alterations in PI3K-AKT-mTOR pathway, the lipid markers elected, consequently, are part of this process during embryogenesis, as the mTOR signaling pathway is active in both neurogenesis and autophagy signaling processes (Figure 4). The latter is triggered as protection against infections, as discussed before, and, during a neurogenesis process, autophagy activation may lead to neurologic malformations, as in the case of pregnant women infected by ZIKV.

The last marker described in this contribution for understanding the metabolomics of ZIKV infection is a ganglioside, GM2 [m/z 1323.7423], which belongs to the class of sphingolipids. These lipids are known for their relation with the identification and inclusion of several types of viruses into the cells, as soon as the infection process begins, as in the case of polyomavirus and HIV (Mazzon and Mercer, 2014). Gangliosides are located in the external side of the plasma membrane and regulate cell development processes (Coskun et al., 2011); additionally, as they are part of the membrane's outer layer, these molecules are explored by pathogens, functioning as binders in the process of cell recognition and supporting endocytosis of microbes (Eidels et al., 1983; Tsai et al., 2003). These lipids are also fundamental for viral genome replication, where they compose the viral replication complex (VRC) in conjunction with NS4A (Wang et al., 2016), as demonstrated in a study with DENV, where NS4A, is responsible for anchoring the VRC in the endoplasmic reticulum. Thus, together, gangliosides and NS4A are essential molecules for viral replication. In addition, gangliosides are further associated with an important complication attributed to infections: the Guillain-Barre Syndrome (GBS), an autoimmune condition where the host's immune system attacks the gangliosides of neurons. This clinical picture has been described in a series of infections (Cao-Lormeau et al., 2016), including ZIKV (Kuwabara and Yuki, 2013). Our results, therefore, suggest that the elected ganglioside is related to GBS, as these molecules are related with the formation of viral replication vacuoles from plasma membrane invaginations in the infected cells (Wang et al., 2016); because of its location in the plasma membrane, this lipid is subject to recognition by the immune system and works as a marker for infected cells. However, as neurons effectively present this molecule under normal circumstances, the immune system attacks not only the infected cells, but the whole environment, due to cross-identification (Kuwabara and Yuki, 2013; Liang et al., 2016; van Doorn and Jacobs, 2016).

Our results suggest, ultimately, that it is possible to perform a viral infection mechanism study through the direct analysis of the serum from infected patients. All biomarkers were elected and validated by statistical analysis, and are in consonance with previous studies that were focused on proteins (kinases and phosphorylases) involved in the infection process, whilst the biomarkers presented herein are substrates/products of these enzymes. Studies on viral infections, such as DENV and H5N1 have explored the inhibition of ACE (Tikellis et al., 2011; Zou et al., 2014; Hernández-Fonseca et al., 2015; Gu et al., 2016) and kinases involved in the PI3K-AKT-mTOR pathway (Easton et al., 2005; Tokuda et al., 2010; Liang et al., 2016); seven out of the eight biomarkers reported in this contribution are directly related to these enzymes, corroborating the relevance of these molecules and providing the targets in which substrate they work. Therefore, the metabolomic insight on human infection by ZIKV provided by this contribution broadens the knowledge of the pathophysiological aspects of the disease by elucidating molecular targets of the cell immune response facing viral infection and replication; this also provides grounds for further developments within the field of pharmacology for differential therapies, interventions and insights in ZIKV infection management.

## Author contributions

CM and JD performed sample collection, experiments, and wrote the manuscript. DdO, TG, CE, EL, and VP performed data analysis and performed manuscript review. RC idealized all experiments and managed the research group. The Zika Unicamp Network is mentioned as an initiative from the University of Campinas of mutual collaboration in the Brazilian Plan for Fighting Zika Virus.

### Conflict of interest statement

The authors declare that the research was conducted in the absence of any commercial or financial relationships that could be construed as a potential conflict of interest.
