# Corrigendum: Serum Metabolic Alterations upon Zika Infection

**DOI:** 10.3389/fmicb.2017.02373

**Published:** 2017-11-29

**Authors:** Carlos F. O. R. Melo, Jeany Delafiori, Diogo N. de Oliveira, Tatiane M. Guerreiro, Cibele Z. Esteves, Estela de O. Lima, Victoria Pando-Robles, Rodrigo R. Catharino

**Affiliations:** ^1^INNOVARE Biomarkers Laboratory, School of Pharmaceutical Sciences, University of Campinas, Campinas, Brazil; ^2^Unidad de Proteómica, Instituto Nacional de Salud Publica, Cuernavaca, Mexico

**Keywords:** Zika virus, mass spectrometry, metabolomics, viromics

In the original article, there was a mistake in the legend for Figure 4 as published. The text in the published legend reads that Ang I or II, Ang 1-9, and Ang 1-7 are signaling to *activate* the autophagy process, where it should be *inhibiting* the process; in addition, the legend states that the inhibition of AKT by viral proteins culminates in the *inhibition* of autophagy, where it should be *activating* it. The correct legend appears below.

**Figure 4:**
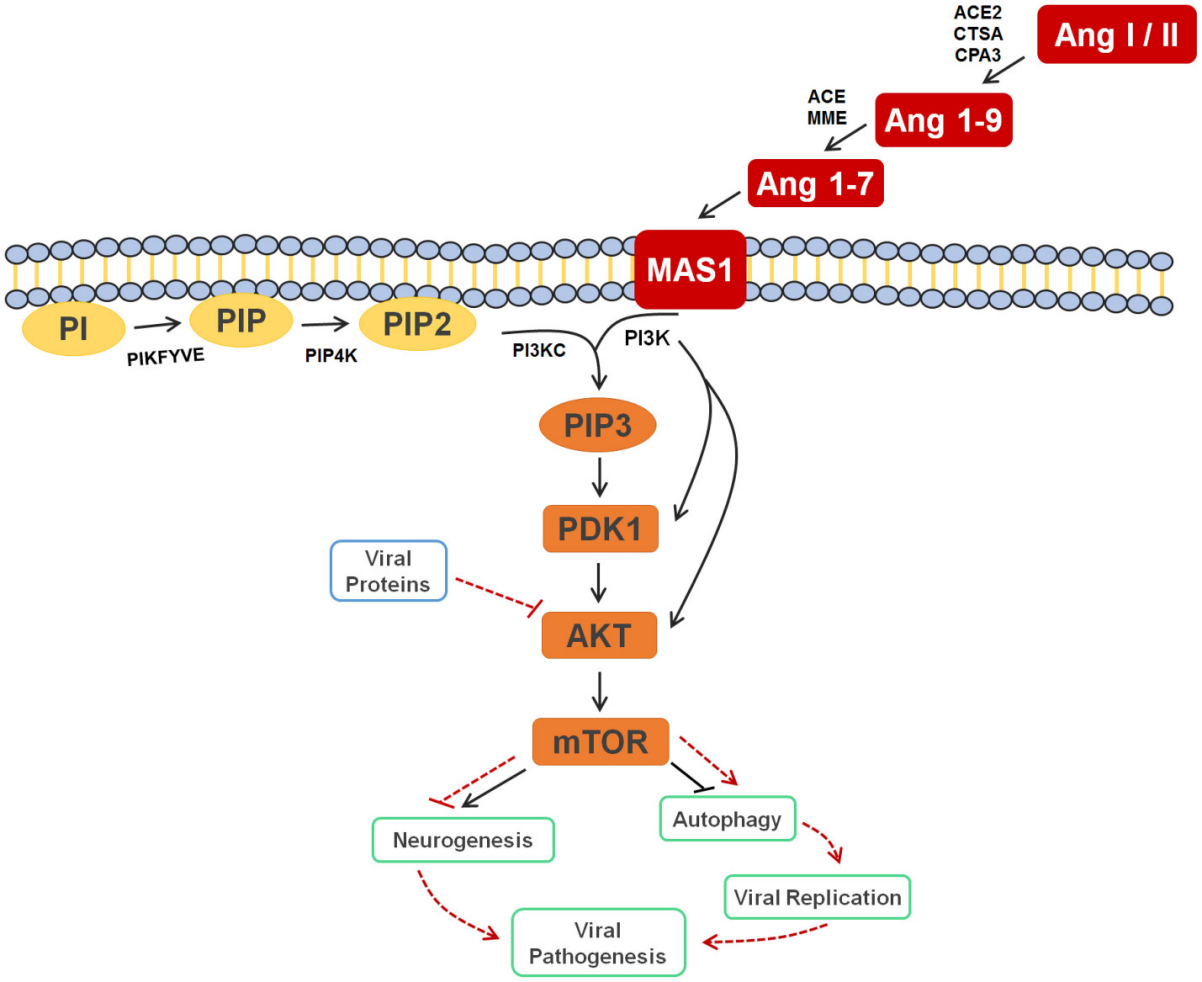
Cell signaling pathway scheme of metabolic alterations due to Zika virus infection. The scheme shows the cell response, attempting to control the viral infection, with Ang I or II, Ang 1-9 and Ang 1-7 signaling to inhibit autophagy process, which would lead to decreased viral replication. It is also possible to see the close participation of lipids PI, PIP2 and PIP3 as key players in this process, all of which were elected as biomarkers. The scheme also shows the inhibition of AKT by the viral proteins of ZIKV (solid and dashed red lines), which culminates in the activation of autophagy, so that replication can occur. In a parallel mechanism, it is possible to see that the same pathway is responsible for the inhibition of neurogenesis. Ang I / II, Angiotensin I / II; Ang 1-9, Angiotensin 1-9; Ang 1-7, Angiotensin 1-7; MAS1, MAS receptor; PI, 1-Phosphatidyl-D-myo-inositol; PIP, Phosphatidylinositol 5-phosphate; PIP2, Phosphatidylinositol-4,5-bisphosphate; PIP3, Phosphatidylinositol-3,4,5-trisphosphate; PDK1, 3-phosphoinositide-dependent protein kinase 1; AKT, AKT serine/threonine kinase 3; mTOR, mechanistic target of rapamycin (atypical serine/threonine kinase); PIKFYVE, 1-phosphatidylinositol-3-phosphate 5-kinase; PIP4K, phosphatidylinositol-5-phosphate 4-kinase type 2 alpha; PIK3C, phosphoinositide-3-kinase regulatory subunit 5; PIK3, phosphoinositide-3-kinase regulatory; ACE2, angiotensin-converting enzyme 2; CTSA, carboxypeptidase C; CPA3, carboxypeptidase A3; ACE, angiotensin-converting enzyme; MME, Neprilysin.

Figure 4. Cell signaling pathway scheme of metabolic alterations due to Zika virus infection. The scheme shows the cell response, attempting to control the viral infection, with Ang I or II, Ang 1-9 and Ang 1-7 signaling to inhibit autophagy process, which would lead to decreased viral replication. It is also possible to see the close participation of lipids PI, PIP2 and PIP3 as key players in this process, all of which were elected as biomarkers. The scheme also shows the inhibition of AKT by the viral proteins of ZIKV (solid and dashed red lines), which culminates in the activation of autophagy, so that replication can occur. In a parallel mechanism, it is possible to see that the same pathway is responsible for the inhibition of neurogenesis. Ang I / II, Angiotensin I / II; Ang 1-9, Angiotensin 1-9; Ang 1-7, Angiotensin 1-7; MAS1, MAS receptor; PI, 1-Phosphatidyl-D-myo-inositol; PIP, Phosphatidylinositol 5-phosphate; PIP2, Phosphatidylinositol-4,5-bisphosphate; PIP3, Phosphatidylinositol-3,4,5-trisphosphate; PDK1, 3-phosphoinositide-dependent protein kinase 1; AKT, AKT serine/threonine kinase 3; mTOR, mechanistic target of rapamycin (atypical serine/threonine kinase); PIKFYVE, 1-phosphatidylinositol-3-phosphate 5-kinase; PIP4K, phosphatidylinositol-5-phosphate 4-kinase type 2 alpha; PIK3C, phosphoinositide-3-kinase regulatory subunit 5; PIK3, phosphoinositide-3-kinase regulatory; ACE2, angiotensin-converting enzyme 2; CTSA, carboxypeptidase C; CPA3, carboxypeptidase A3; ACE, angiotensin-converting enzyme; MME, Neprilysin.

In the original article, there was a mistake in Figure 4 as published. The published figure erroneously presented a solid black arrow pointing to autophagy and a dashed red line blocking the same pathway, when it should have been otherwise: a solid black line blocking and a dashed red arrow pointing. In addition, the figure was updated with dashed red lines and arrows to point out mechanisms elicited by ZIKV. The corrected Figure 4 appears below.

In the original article, there was an error. During the review process, Figure 4 was updated erroneously as per the corrections above, triggering a chain of errors, where we stated that the autophagy process is blocked upon ZIKV infection; since it is the contrary, i.e., autophagy process is favored upon ZIKV infection, some other sections were erroneously updated in the review. This corrigendum stated that the correct discussion is that autophagy is BLOCKED under the activation of the AKT/mTOR pathways by the renin-angiotensin system, and ACTIVATED through mTOR blockade by ZIKV proteins.

A correction has been made to the Abstract and the corrected paragraph should be:

Zika virus (ZIKV) infection has recently emerged as a major concern worldwide due to its strong association with nervous system malformation (microcephaly) of fetuses in pregnant women infected by the virus. Signs and symptoms of ZIKV infection are often mistaken with other common viral infections. Since transmission may occur through biological fluids exchange and coitus, in addition to mosquito bite, this condition is an important infectious disease. Thus, understanding the mechanism of viral infection has become an important research focus, as well as providing potential targets for assertive clinical diagnosis and quality screening for hemoderivatives. Within this context, the present work analyzed blood plasma from 79 subjects, divided as a control group and a ZIKV-infected group. Samples underwent direct-infusion mass spectrometry and statistical analysis, where eight markers related to the pathophysiological process of ZIKV infection were elected and characterized. Among these, Angiotensin (1-7) and Angiotensin I were upregulated under infection, showing an attempt to BLOCK autophagy of the infected cells. However, this finding is concerning about hypertensive individuals under treatment with inhibitors of the Renin-Angiotensin System (RAS), which could reduce this response against the virus and exacerbate the symptoms of the infection. Moreover, one of the most abundant glycosphingolipids in the nervous tissue, Ganglioside GM2, was also elected in the present study as an infection biomarker. Considered an important pathogen receptor at membrane's outer layer, this finding represents the importance of gangliosides for ZIKV infection and its association with brain tropism. Furthermore, a series of phosphatidylinositols were also identified as biomarkers, implying a significant role of the PI3K-AKT-mTOR Pathway in this mechanism. Finally, these pathways may also be understood as potential targets to be considered in pharmacological intervention studies on ZIKV infection management.

**Discussion, paragraph 2:**

Given that Ang (1-7) diminishes the severity of pathogen infections due to alterations in the cell machinery, thereby breaking its life cycle (Saraiva et al., 2011; Fedson, 2016), the biomarkers elucidated in our study reveal that these species may also be linked to the control of the immune response to ZIKV infection. Although one of the most primordial forms of innate immune defense may be autophagy, which has been described as a mechanism involved with antigen presentation, microbe elimination, and secretion of immune mediators, it is also a response involved in viral replication (Tallóczy et al., 2006; Deretic et al., 2013). The Ang (1-7) signaling pathway, for instance, is also related to inhibition of the process of autophagy, a process that favors the formation of the viral replication complex, thus decreasing viral replication rate in the organism (Saraiva et al., 2011; Petersen et al., 2016). Electing both Ang I and Ang (1-7) as ZIKV group biomarkers, therefore, allow us to infer that the RAS is part of the immune response process against ZIKV in humans.

**Discussion, paragraph 4:**

This is the first time that lipids for ZIKV infection are described, whereas the great majority of previous contributions deal with general immune response species such as proteins, as well as with molecules potentially linked with microcephaly (Petersen et al., 2016). Taking into account that neurologic malformations such as microcephaly are associated with alterations in PI3K-AKT-mTOR pathway, the lipid markers elected, consequently, are part of this process during embryogenesis, as the mTOR signaling pathway activates neurogenesis and inhibits autophagy signaling processes (Figure 4). The latter is blocked as protection against infections, as discussed before, and, during a neurogenesis process, autophagy activation may lead to neurologic malformations, as in the case of pregnant women infected by ZIKV.

The authors apologize for these errors and state that this does not change the scientific conclusions of the article in any way.

## Conflict of interest statement

The authors declare that the research was conducted in the absence of any commercial or financial relationships that could be construed as a potential conflict of interest.
